# Microbiota Modulation as an Approach to Prevent the Use of Antimicrobials Associated with Canine Atopic Dermatitis

**DOI:** 10.3390/biomedicines13102372

**Published:** 2025-09-27

**Authors:** Tânia Lagoa, Luís Martins, Maria Cristina Queiroga

**Affiliations:** 1 MED—Mediterranean Institute for Agriculture, Environment and Development & CHANGE-Global Change and Sustainability Institute, Universidade de Évora, Polo da Mitra, Ap. 94, 7006-554 Évora, Portugal; d50889@alunos.uevora.pt (T.L.); lmlm@uevora.pt (L.M.); 2 IIFA—Institute for Advanced Studies and Research, Universidade de Évora, Polo da Mitra, Ap. 94, 7006-554 Évora, Portugal; 3 School of Biosciences of Elvas, Polytechnic Institute of Portalegre, 7350-092 Elvas, Portugal; 4 Department of Veterinary Medicine, School of Sciences and Technology, Universidade de Évora, Polo da Mitra, Ap. 94, 7006-554 Évora, Portugal

**Keywords:** gut microbiota, dysbiosis, atopic dermatitis, antimicrobial resistance, diet, probiotics, prebiotics, postbiotics, fecal microbiota transplantation

## Abstract

Modern lifestyle factors—such as dietary changes, reduced microbial exposure, and genetic susceptibility—profoundly influence the composition and function of the commensal microbiota. Additionally, dysregulation of the gut microbiota has been linked to impaired immune responses and an increased prevalence of skin disorders, including allergies and inflammatory conditions, thereby underscoring the importance of the gut–skin axis. Chronic gastrointestinal and dermatological manifestations frequently lead to excessive antimicrobial use, which in turn fosters the selection and colonization of multidrug-resistant organisms, most notably methicillin-resistant *Staphylococcus pseudintermedius* (MRSP) in companion animals. Furthermore, the growing threat of antimicrobial resistance (AMR) to both human and animal health reinforces the urgent need for alternative strategies like modulating the gut microbiota, which also contributes to the prevention and management of skin conditions. Against this backdrop, the present review aims to evaluate microbiota modulation as an alternative and complementary approach to antimicrobial therapy, focusing on its ability to restore microbial balance, strengthen epithelial barrier integrity, and improve overall health outcomes in dogs affected by atopic dermatitis (cAD). Promising interventions—including probiotics, prebiotics, postbiotics, and fecal microbiota transplantation—are highlighted for their potential role in mitigating AMR and warrant further investigation as sustainable therapeutic strategies.

## 1. Introduction

The term microbiota refers to the community of microorganisms inhabiting a specific ecosystem, such as the oral cavity, gastrointestinal tract, vaginal environment, or skin. This complex community comprises thousands of bacterial species, viruses, fungi, and some eukaryotic organisms [1,2]. The collective genetic material of these microorganisms is referred to as the microbiome [1]. The microbiota depends on genetic factors, diet, and exposure to environmental conditions [3,4]. In contemporary urban settings, both humans and companion animals, such as dogs, are increasingly subjected to environmental pollutants, elevated levels of indoor allergens, and reduced exposure to natural outdoor environments. Concurrently, demographic shifts, such as smaller household sizes and decreased interaction with diverse microbial environments during early life, which reduce the window of opportunity for the immune system education, have contributed to a decline in microbial diversity encountered by the host [5,6,7]. This trend is frequently associated with the hygiene hypothesis, which suggests that reduced microbial exposure, a hallmark of Westernized lifestyles, may disrupt the normal development of the immune system. Limited interaction with commensal and environmental microorganisms is believed to impair the establishment of immunological tolerance and homeostasis, potentially increasing susceptibility to immune-mediated disorders [6,7,8,9,10].

The International Committee on Allergic Diseases of Animals (ICADA) recently concluded that an updated definition of canine atopic dermatitis (cAD) was necessary, reflecting the most recent advances in understanding its pathogenesis. cAD is a hereditary, T cell-mediated, inflammatory, and pruritic disease with a multifactorial pathogenesis involving a complex interplay of genetic and environmental factors. Allergen exposure contributes to skin barrier dysfunction, immune dysregulation, and dysbiosis of the cutaneous microbiota [11,12,13]. Clinical signs typically develop between 1 and 3 years of age, with pruritus as the primary symptom, often accompanied by erythema, conjunctivitis, otitis, and skin lesions, all of which compromise the well-being of both pets and their owners. Prevalence varies geographically, with predisposed breeds, including the Boxer, Bulldog, Labrador Retriever, Pug, and West Highland White Terrier. Common allergens associated with cAD include house dust mites (the most prevalent), as well as molds and pollens from weeds, grasses, and trees [13].

Environmental factors and dietary patterns are also known to influence gene expression and shape the composition of the gut microbiota. In turn, alterations in gut microbial communities may contribute to skin disorders—or conversely, skin disorders may influence gut composition—a bidirectional relationship commonly referred to as the “gut–skin axis.” This interaction has become a crucial field of investigation for understanding skin health and disease. In this context, lifestyle factors such as diet, living environment, and daily interactions with the surroundings can profoundly modulate the microbiota and influence the balance between health and disease, a topic that has attracted increasing scientific interest in recent years [14,15,16].

The aim of this review is to examine microbiota modulation as a preventive strategy to reduce reliance on antimicrobials, highlighting its potential to maintain or restore microbial homeostasis and support host health in cAD. Given the scarcity of large-scale veterinary studies on this topic, some evidence is extrapolated from human research to provide broader insights into possible applications in companion animals [17].

## 2. The Gastrointestinal Tract and Host Immune System

The gastrointestinal (GI) tract is a vital organ constantly exposed to a wide range of dietary components and potentially harmful microorganisms. It is lined by a mucosal surface covered by simple columnar epithelium composed of intestinal epithelial cells (IECs). Just beneath this epithelial layer lies the largest reservoir of immune cells in the body, forming the foundation of mucosal immunity [18]. IECs, which are non-hematopoietic in origin, serve both as a physical and immunological barrier. Beyond their structural role, they actively participate in immune regulation by producing cytokines, chemokines, hormones, and immune-regulatory molecules. IECs also express carbohydrate moieties and the secretory component of immunoglobulin A (SIgA), which is synthesized by plasma cells, thereby contributing to antigen exclusion and immune homeostasis [19]. Another crucial component of defense is the mucus layer, produced by Goblet cells and composed mainly of mucins. This layer acts as a frontline defense by limiting microbial translocation and maintaining spatial separation between the microbiota and the epithelium [18,20]. The gut-associated lymphoid tissue (GALT) forms the core of the innate and adaptive immune system in the gut. The GALT maintains a delicate balance between inducing tolerance to dietary antigens and commensal microorganisms, while mounting effective immune responses against pathogenic threats [21]. These frequent interactions between immune cells and antigens trigger continuous immune activity, including the development of germinal centers (GCs), which in turn influence both the composition of the commensal microbiota and the immune reactions to invading pathogens [22]. Innate immunity includes pattern recognition receptors (PRRs), antimicrobial peptides, and phagocytes, while the adaptive arm involves T and B lymphocytes capable of antigen-specific responses. One of the most influential environmental modulators of this immune landscape is the intestinal microbiota. It contributes to immune system development and modulates allergen tolerance, particularly through interactions with regulatory T (Treg) cells [22]. The microbiota also ferments indigestible polysaccharides, producing short-chain fatty acids (SCFAs) such as butyrate, acetate, and propionate which support epithelial integrity, reduce intestinal permeability, and exert epigenetic and post-translational effects on host cells [9,20]. Importantly, the microbiota plays a central role in shaping innate immune memory, especially during early life [5]. This form of non-specific memory represents a co-adaptive evolutionary mechanism, allowing the innate immune system to mount faster responses to pathogens [23]. However, this memory can also become detrimental in conditions such as chronic inflammation or sepsis, where persistent activation may lead to systemic immune dysfunction, including tissue damage, exhaustion, or immune paralysis [24].

Commensal bacteria, particularly lactic acid bacteria, produce metabolites that reduce levels of inflammatory cytokines and enhance epithelial barrier function. Among these, SCFAs are the most abundant, together with B-group vitamins and other methyl-group donors metabolized by the gut microbiome [8,25]. Dysbiosis, characterized by reduced microbial abundance and altered function, predisposes to disruption of the epithelial barrier, enabling the access of toxins and allergens into the host [26]. Consequently, circulating immunoglobulin E (IgE) and basophil levels increase, predisposing individuals to the development of allergies and chronic conditions such as atopic dermatitis (AD). In dogs, atopic individuals exhibit significantly lower gut microbiota diversity, loss of beneficial microorganisms and overgrowth of pathogens, compared to healthy controls, as will be discussed in more detail in the following sections [9,27]. Overall, the interplay between the intestinal epithelium, mucosal immune components, mucus barrier, and the microbiota is essential for maintaining homeostasis, ensuring pathogen defence, and preserving tolerance to non-threatening stimuli.

### 2.1. The Intestinal Microbiota and Dysbiosis

The collection of microorganisms residing in the gastrointestinal tract is called the gut flora or microbiota. It represents the largest source of non-self-antigens in the body and is composed of various microbes, including bacteria, viruses, fungi, and protozoa. Among these, bacteria are the most abundant, primarily strict or facultative anaerobes, with Firmicutes, Fusobacteria, and Bacteroidetes being the predominant phyla [28]. The complexity of the microbiota has historically been underestimated using traditional culture techniques.

As mentioned above, the resident microbiota plays a crucial role in maintaining the structural and functional integrity of the gut as well as in regulating the immune system. It is an important driver of host immunity, helps protect against invading enteropathogens, and provides nutritional benefits to the host [29]. Variations in microbiota diversity, structure, and function—including changes in bacterial metabolites—are referred to as dysbiosis. In dogs, dysbiosis resulting from intestinal inflammation or antibiotic use can reduce the abundance of *Clostridium hiranonis*, a microorganism responsible for converting primary to secondary bile acids. This disruption may favor the overgrowth of *Clostridioides (Clostridium) difficile* [28].

Khosravi et al. report that commensal microorganisms support the maintenance of both hematopoietic stem cell (HSC)-derived and embryo-derived myeloid cells under steady-state conditions. In the absence of commensal microbiota, significant defects are observed in multiple innate immune cell populations, including neutrophils, monocytes, and macrophages in peripheral tissues [30]. By influencing the differentiation pathways of innate immune cells, the gut microbiota equips the host to mount effective immune responses upon encountering pathogens. In contrast, germ-free or antimicrobial-treated mice show impaired clearance of systemic bacterial infections. These findings demonstrate that gut microorganisms have co-evolved with the host to actively regulate immune homeostasis at its foundation—through the modulation of hematopoiesis [30]. Additionally, commensal microbiota produces bacteriocins that selectively inhibit the growth of bacterial strains and antimicrobial-resistant pathogens, while also promoting cellular proliferation, mucus secretion, villus thickening, vascularization, and maintenance of epithelial junctions [31,32].

Short-chain fatty acids, produced by some microorganisms in the microbiota, are saturated aliphatic organic compounds containing one to six carbon atoms, with acetate (C2), propionate (C3), and butyrate (C4) making up approximately 95% of the total SCFAs produced, which are essential for nourishing enterocytes, possess anti-inflammatory properties, and help modulate immune response [33] and create an acidic intestinal milieu, which suppresses the proliferation of pathogenic microorganisms [34]. Furthermore, Becattini et al. reported that SCFAs prevented lung allergic inflammation by promoting the development of phagocytes with reduced Th2-polarizing capacity [32].

The gut microbiota and their metabolic products can be detected by Toll-like receptors (TLRs), whose interactions with microbes play a key role in maintaining systemic immune balance [34]. Besides, the gut microbiota influences the host’s ability to generate adaptive immune responses by promoting myelopoiesis, specifically through the expansion of macrophages and neutrophils in the spleen [35]. Moreover, the gut microbiota has been shown to influence the outcomes of vaccines and the effectiveness of anticancer therapies [22].

In addition to immune modulation, the gut microbiota plays a critical role in nutrient metabolism, including the breakdown of dietary fiber and complex carbohydrates that the human host and certain animal species cannot digest independently and its essential role in the production of certain nutrients such as vitamin K [15]. Importantly, gut microorganisms are also involved in the regulation of brain development, neurotransmission, and behavior—collectively known as the “gut-brain axis”—which may contribute to the onset and progression of various neurodevelopmental, neuropsychiatric, and neurological disorders [36].

However, some microbes in the gut, known as pathobionts, have the potential to cause disease under certain conditions. While usually present in low numbers and harmless in healthy individuals, their overgrowth can lead to illness—representing a form of dysbiosis. A well-documented example of this is the increased presence of *Proteobacteria*, especially the *Enterobacteriaceae* family, which includes *E. coli*, *Shigella*, and *Klebsiella* [37] and enterocolitis in humans and horses caused by *Clostridioides (Clostridium) difficile* [38,39].

Disruption of gut microbiota, also known as dysbiosis, may impair immune system homeostasis through altered production of microbial metabolites, such as SCFAs and bile acids (BAs) [40]. This imbalance can foster a pro-inflammatory microenvironment, engaging immune components that facilitate the transcription of type I interferons. These interferons, in turn, stimulate the maturation of invariant natural killer T (iNKT) cells [35], and contribute to the onset of severe health conditions that affect both the gastrointestinal tract and extra-intestinal organs [8,14,41].

Disruptions in the gut microbiota may also allow microbes and their metabolites to enter the bloodstream, influencing disease symptoms in distant organs such as the brain, liver, kidneys, lungs, and skin [34].

Therefore, maintaining a diverse and healthy gut microbiota is fundamental, not only for overall health but also to minimize the use of antimicrobials and thus as a strategy to combat antimicrobial resistance (AMR).

### 2.2. The Role of Intestinal Microbiota in Atopic Dermatitis and Other Skin Conditions

**Figure 1:**
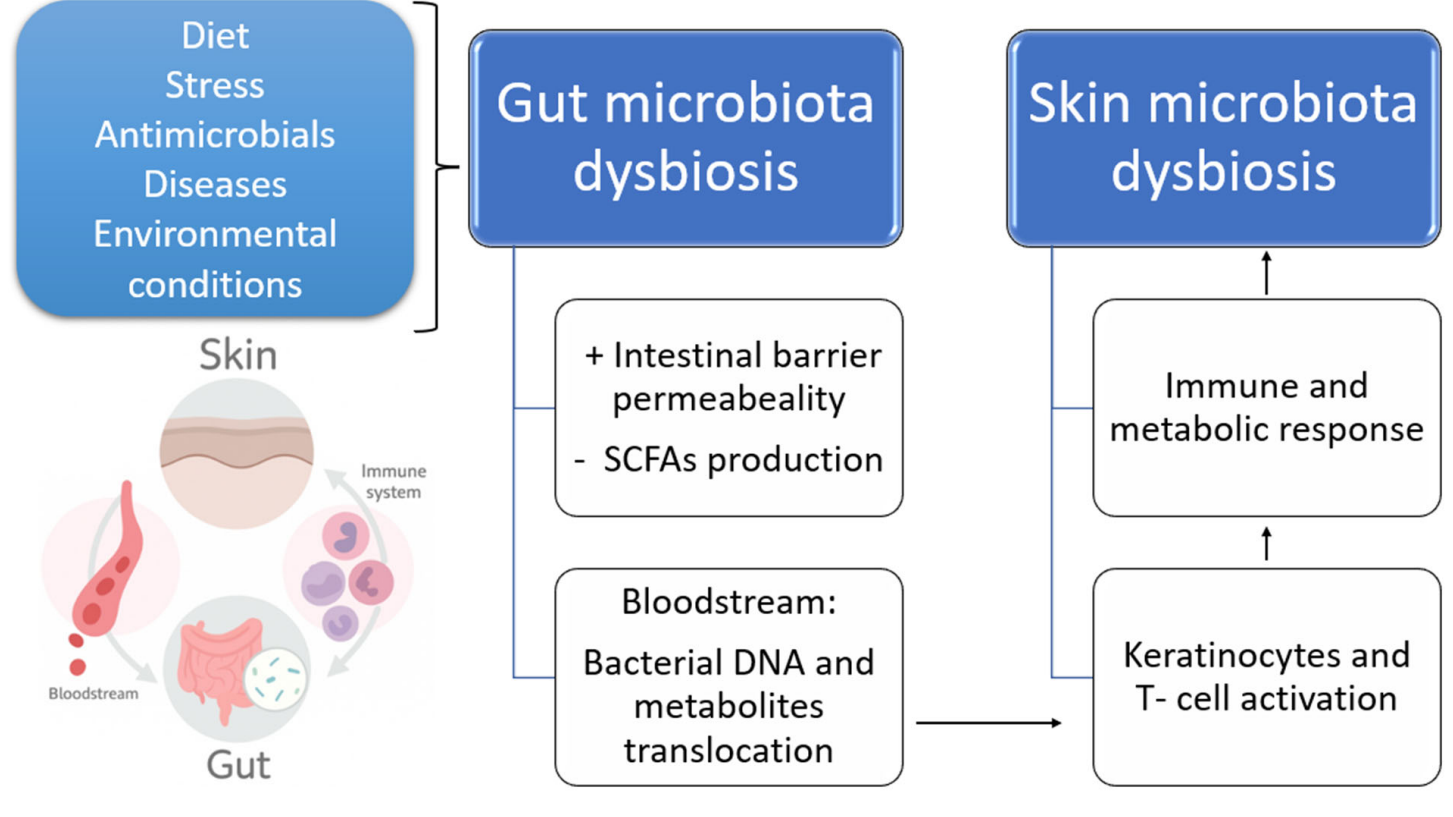
Schematic representation of the gut–skin axis. Various factors such as diet, stress, antimicrobials, diseases, and environmental conditions can disrupt gut microbiota homeostasis, leading to intestinal dysbiosis. This dysbiosis increases intestinal barrier permeability, changes SCFA production, and enables the translocation of bacterial DNA and metabolites into the bloodstream. These changes trigger systemic immune and metabolic responses, including keratinocyte and T-cell activation, ultimately contributing to skin microbiota dysbiosis and skin inflammation. +: increased, -: decreased.

Both the skin and gut are dynamic, complex immunological and neuroendocrine organs that are constantly exposed to the external environment and interact with the host’s diverse microbial communities. Numerous studies have reported gut and skin dysbiosis associated with various dermatological conditions, including AD, psoriasis, rosacea, and acne. Evidence increasingly supports a functional connection between the gut and skin—referred to as the gut–skin axis—mediated by the microbiota, which plays a critical role in modulating immune responses and maintaining epithelial barrier integrity [42,43]. When the skin barrier is disrupted by injuries, burns, cuts, or chronic diseases, microorganisms can penetrate into deeper skin layers. In such conditions, the skin’s protective capacity is diminished, allowing normally harmless commensal microorganisms to cause infections [44]. Wang et al. conducted a study in China involving 722 canine samples from bacterial skin infections, where they isolated microbial agents commonly associated with canine dermatological infections, including *Staphylococcus pseudintermedius*, *Pseudomonas aeruginosa*, and *E. coli* [45]. These pathogens are frequently implicated in chronic or complicated skin conditions, often reflecting underlying systemic diseases or compromise of the cutaneous barrier. The intestinal and epidermal barriers are interconnected via the systemic circulation (blood and lymph), enabling bidirectional communication. Dysbiosis is associated with dysfunction of both barriers. In the gut, this includes microbial imbalance, weakened mucus and IgA defenses, increased permeability, and inflammation. In the skin, it is characterized by microbial disruption, reduced production of antimicrobial peptides (AMPs), and impaired keratinocyte function, which together predispose to skin damage (e.g., rashes, thickening) and inflammation. These disturbances are closely linked through immune dysregulation, particularly involving Th2-mediated responses [10]. In summary, the integrity of the intestinal barrier—supported by the protective functions of mucus, immune cells, IgA, AMPs produced by epithelial cells, and GALT—prevents the translocation of gut bacteria into the bloodstream, thereby contributing to the maintenance of skin homeostasis [42] (Figure 1). Epidemiologic surveys found that patients with moderate to severe atopic dermatitis had higher levels of *C. difficile*, *E. coli* and *Staphylococcus aureus* in their gut microbiota, whereas healthy patients or those with mild AD had higher levels of *Pneumococci*. Moreover, patients with AD have a decrease of beneficial microorganisms, such as *Lactobacillus* and *Bifidobacterium* [10,46,47].

Atopic dermatitis is a prevalent inflammatory and pruritic dermatological condition, affecting up to 15% of both human and canine populations [9]. The prevalence of atopic dermatitis is increasing globally [25]. Its pathogenesis is multifactorial, involving not only impairment of the cutaneous barrier and immunological dysregulation, but also an increased occurrence of bacterial skin infections, with pyoderma as the most frequent condition, which intensifies the use of antimicrobials, increasing the risk of antimicrobial resistance (AMR) and is also associated with alterations in both the skin and gut microbiota [9,41,45].

Differences in the fecal microbiota have been demonstrated between patients with cAD and healthy control individuals (Table 1) [9,14,45,48,49]. Furthermore, Rostaher et al. proposed that dogs with atopic dermatitis exhibit a reduced gut microbiota diversity compared to healthy counterparts, although large-scale studies are needed to confirm this observation (Table 1) [9]. Thomsen et al. clearly demonstrated dysbiosis in both the skin and gut microbiota of Shiba Inu dogs with AD. The study revealed that *Clostridium sensu stricto* and *Escherichia*/*Shigella* were significantly increased in AD-affected dogs compared to healthy controls; however, these microbial alterations were largely reversed following oclacitinib treatment (Table 1) [49]. Other authors investigated the oral and gut microbiome of healthy dogs and those with AD using 16S rRNA gene amplicon sequencing in a purebred Shiba Inu colony. Analysis of microbial diversity, differential abundance, and co-occurrence patterns revealed that atopic dermatitis is associated with significant alterations in both the oral and gut microbiota of affected dogs [50].

In humans, Song et al. collected fecal samples from patients with AD and found decreased levels of butyrate and propionate, both of which are SCFAs with anti-inflammatory properties. This reduction is likely due to an intraspecies compositional shift in *Faecalibacterium prausnitzii*, leading to a decline in strains that produce high amounts of SCFAs, such as the A2-165 strain. A deficiency of this strain has also been associated with Crohn’s disease [51]. Bull et al. characterized the gut microbiota of one-month-old infants with atopic eczema and observed significantly reduced bacterial diversity, particularly within the *Bacteroides* phylum, compared to infants without the condition [52]. In contrast, studies by Liu et al. in both children and adults with AD reported an enrichment of *Parabacteroides* spp. and *Bacteroides* spp. in individuals with severe disease relative to those with mild forms [53]. These findings suggest that certain microbial shifts may be associated with disease severity and immune dysregulation. *Bacteroides* spp. are known to mediate T-cell activation and include strains capable of causing serious skin and soft tissue infections. Notably, both *Parabacteroides* spp. and *Bacteroides* spp. produce sphingolipids—bioactive lipids that play critical roles in regulating inflammation and immunity by activating natural killer T (NKT) cells and inducing rapid cytokine release. Consistent with this, patients with AD often exhibit altered sphingolipid profiles, particularly reduced ceramide levels, which contribute to skin barrier dysfunction [54]. These lipid alterations may also be exploited by pathogens such as *Clostridium botulinum* and *Vibrio cholerae*, enhancing their virulence and further compromising host defense [53]. Other studies revealed that the presence of clostridia in the gut at 5 and 13 weeks of age has been linked to a higher likelihood of developing AD during the following six months [55]. Patumcharoenpol et al. identified higher amounts of key gut bacterial genera associated with SCFAs production, such as species of *Anaerostipes*, *Butyricicoccus*, *Ruminococcus*, and *Lactobacillus*, in children with AD [56]. Conversely, Sasaki et al. demonstrated that, at 360 days of age, children with a high abundance of *Ruminococcus bromii*—a specialized primary starch degrader—had increased levels of butyrate and a markedly lower prevalence of AD compared to those with low *R. bromii* abundance (11.1–12.5% vs. 44.4–52.5%). However, this association was independent of the presence of other major butyrate-producing bacterial taxa [57].

Xue et al. demonstrated the bidirectional immunological communication between the gastrointestinal tract and the skin. Their study showed that *Helicobacter pylori* infection attenuated calcipotriol-induced AD symptoms by promoting the expansion of CD4^+^CD25^+^Foxp3^+^ regulatory T cells (Tregs) in peripheral blood. This immunomodulatory effect was associated with increased expression of the anti-inflammatory cytokines IL-10 and TGF-β, along with a reduction in the pro-inflammatory cytokine IL-4. These findings suggest a potential protective role of *H. pylori* against AD, supporting the existence of a gut–skin axis in immune regulation [58]. Recent studies have provided evidence that skin exposure to external factors—such as ultraviolet (UV) radiation [59] or topical medications, like imiquimod (IMQ), a Toll-like receptor 7 agonist [60], and balneotherapy [61]—can influence the composition of the gut microbiota. Boosting vitamin D levels through increased skin synthesis via phototherapy may serve as a potential supportive therapy for inflammatory bowel disease. This approach could also benefit from the gentle systemic immunosuppressive effects of UV exposure on the skin [62].

**Table 1:** Studies conducted on the microbiota of DA and healthy individuals through 16S rRNA sequencing.

Patient	N	Site	Microbiota						Ref.
			AD			Healthy			
			Diversity	Increased	Decreased	Diversity	Increased	Decreased	
Adult Beagle dog	7	Gut	−	*Conchiformibius*, *Catenibacterium* spp. *Ruminococcus gnavus* group *Megamonas*	*Lachnospira* *Ruminococcus torques* group *Anaerovocacaceae* (family) *Peptostreptococcaceae*/*Peptoclostridium* UCG—005 (*Oscillospiraceae*) *Sutterella* *Fusobacterium*	+	*Lachnospiraceae* *Anaerovoracaceae* *Oscillospiraceae* genera *Lachnospira* *Ruminococcus torques* group *Fusobacterium* *Fecalibacterium*		[9]
Dogs	896	Skin	−	*P. aeruginosa* *E. coli* *S. pseudintermedius*		+			[45]
Shiba Inu dogs	40	Gut and skin	−	Skin: *Staphylococcus* *Escherichia/Shigella* *Clostridium sensu stricto*	Gut: *Fusobacteria Megamonas*	+	Gut: *Fusobacteria* *Megamonas* *Bacteroidaceae* family	Skin: *Staphylococcus* *Eschrichia*/*Shigella* *Clostridium sensu stricto*	[49]
Dogs	62	Gut	−	*Streptococcus* spp. *Fusobacterium* spp. *E. coli* *C. difficile*	*Lactobacillus* spp.	+	*L. acidophilus* *Lactobacillus* spp.	*Streptococcus* spp. *Fusobacterium* spp. *E. coli* *C. difficile*	[48]
Shiba Inu Dogs	53	Gut and oral	Oral = Gut	Oral: *Proteobacteria* phylum Gut: *Anaerovoracaceae*	Oral: *Bacteroidota* phylum	+		Gut: *Anaerovoracaceae*	[50]
Human	132	Gut	−	*Faecalibacterium prausnitzii*	*Lactobacillus* *Bifidobacterium* spp.	+		*F. prausnitzii*	[51]
Children	139	Gut	−	*Parabacteroides*	Severe AD (vs. mild): *Clostridium sensu stricto* *Collinsella*	+			[53]
Children	62	Gut	−	*Anaerostipes* *Butyricicoccus* *Ruminococcus* *Lactobacillus* spp.					[56]
Children	70	Gut			*R. broomi*		*R. broomi*		[57]

+: higher, -: lower.

### 2.3. Antimicrobial Treatment and Its Effects on the Intestinal Microbiota

With industrialization, many countries have experienced a substantial increase in the overuse, misuse, and inappropriate prescription of antimicrobials, which has significantly contributed to the emergence and global spread of AMR [31]. Epidemiological data indicate that around 71% of patients in intensive care units undergo antimicrobial treatment, which is also commonly administered in pediatric populations [63,64]. Antimicrobial resistance has become a major public health concern and understanding the multifaceted impact of antimicrobials on gut microbial communities is essential for safeguarding overall human well-being [36]. Maternal antimicrobial therapy during the perinatal period has been associated with gut dysbiosis in the offspring, a condition that may persist up to the end of the first year of life [8]. Mangin et al. collected fecal samples from 31 infants at the end of amoxicillin treatment and assessed bifidobacteria through DNA analysis. The study demonstrated that amoxicillin treatment alters and reduces the population of *Bifidobacterium* spp. in the gut microbiota and increases the risk of obesity, asthma, and allergy development [64]. Buffie et al. demonstrated that even a single administration of clindamycin leads to a substantial and prolonged decline in intestinal microbial diversity, resulting in the sustained depletion of approximately 90% of the native cecal microbiota for up to 28 days [31,65]. Ce Huang et al. [63] employed 16S rRNA gene sequencing to assess the short- and long-term effects of ampicillin, vancomycin, metronidazole, and neomycin on the murine intestinal microbiota. Their findings showed that changes in microbial composition reflected the specific mechanisms of action of each antimicrobial, and that dysbiosis led to increased competition among distinct bacterial communities and thus may reduce diversity and subsequently bile acid production and glucose metabolism [57]. Vrieze et al. demonstrated that vancomycin reduces peripheral insulin sensitivity, and, depending on an individual’s genotype and lifestyle, antimicrobial use may contribute to the development of obesity [66]. Antimicrobial therapy has been shown to compromise immune function by suppressing mitochondrial respiratory activity in immune cells [67]. Also, the development of AMR can compromise immune function, notably through damage to the protective mucin layer. This disruption enables the infiltration of pathogenic microorganisms and hinders the colonization of beneficial commensal bacteria. Additionally, bacterial ability to produce biofilm represents a resistance mechanism that can be selectively enhanced through competitive interactions triggered by antimicrobial-induced selective pressure [68,69]. Such instances of dysbiosis increase the susceptibility of both animals and humans to a range of pathological conditions, including diabetes mellitus, obesity, hepatic dysfunction, cardiovascular diseases, gastrointestinal disorders and neurologic conditions [15]. Antimicrobial-induced disruption of the gut microbiota has been associated with mental health conditions such as anxiety and depression, highlighting the intricate connection between gut and brain health [36].

While antimicrobial administration modifies the composition and variety of the intestinal microbiota, the microbiota itself, in turn, influences the pharmacokinetics and pharmacodynamics of antimicrobials through various mechanisms, altering their absorption, distribution, metabolism and stimulation of an immune response [31]. Certain gut bacterial species are capable of metabolizing beta-lactam antibiotics, thereby inactivating them and reducing their therapeutic efficacy. In experiments, *Bacteroides* strains resistant to ceftriaxone and producing β-lactamase significantly increased the amount of ceftriaxone required to kill a susceptible *Escherichia coli* strain. Thus, β-lactamase production by common gut *Bacteroides* can shield other susceptible commensal bacteria from β-lactam antibiotics [70]. In addition, the gut microbiota can modulate drug distribution by influencing the expression of efflux transporters and drug-metabolizing enzymes within the intestinal epithelium. These modifications can markedly alter the systemic bioavailability and tissue distribution of antimicrobials, ultimately affecting both their effectiveness and potential toxicity, which can promote the development of reduced antimicrobial effectiveness [31]. *Clostridioides* (*Clostridium*) *difficile* infection (CDI) is a known cause of colitis in humans and horses. In both species, disruption of the normal gut microbiota—particularly following antimicrobial therapy and hospitalization—allows toxigenic *C. difficile* strains to proliferate and produce toxins that damage the colon [38,39]. Studies indicate that certain antimicrobials may enhance the expression of adhesin and toxin genes in *C. difficile*, thereby increasing its pathogenic potential [39]. In a study involving 24 horses, *Lactobacillus* spp. were markedly reduced following antibiotic administration. Although responses varied greatly among individuals, antibiotic use consistently altered fecal microbiota composition and function, with effects dependent on the specific drug administered [71].

### 2.4. Antimicrobial Resistance

Another detrimental effect of antimicrobial treatment is selection pressure on antimicrobial-resistant strains. The problem of antimicrobial resistance is a growing concern and deserves the full dedication of scientists to reduce this rate. Surgical procedures are situations that can often result in systemic infections with bacteria from the skin’s microbiota. Developing strategies to reduce these events is the focus of many studies. Vertebroplasty is an example of a minimally invasive technique, which can reduce antimicrobial use and AMR [72]. Other approaches such as preoperative assessment of the skin microbiota and the use of microbiota-friendly antiseptic protocols can help preserve microbial balance and lower infection rates, thereby supporting more judicious antimicrobial use. In addition, post-operative wound management plays a key role in shaping the skin microbiota and influencing infection outcomes. As reviewed by Lagoa et al., the selection of wound dressing materials—particularly those with antimicrobial properties—can directly impact the local microorganism’s environment. While these materials help prevent infection, they may also disturb the native skin microbiome and contribute to AMR if not carefully selected. Therefore, integrating microbiome-preserving approaches into wound care is essential for promoting healing while minimizing resistance risks [73].

In veterinary medicine, this is especially relevant for managing MRSP infections in companion animals. Effective control requires strict hygiene protocols in clinical settings, alongside close monitoring of infection progression—particularly in animals with chronic or predisposing conditions. Additionally, surveillance of bacterial strain dynamics and early detection of resistance trends are essential to guide targeted interventions [45].

## 3. Microbiota Manipulation

Microbiota manipulation has gained attention for its potential in managing both skin and gut infections. Furthermore, modulating the composition and function of the gut microbiota represents a promising strategy to reduce the development and transmission of AMR, offering a systemic and preventive approach to infectious disease management in both human and veterinary medicine [43,74,75].

### 3.1. Diet

The microbiota is highly responsive to various dietary inputs, which significantly influence its composition and functional activity [32]. Several studies identified differences in the gut microbiota of dogs fed with different diets. The effect of diet on microbiota can occur rapidly, on the order of days, or be long-lasting over years and even generations [76].

Some commercial dog foods are formulated with cereals such as wheat, rye, and barley, which contain gluten or structurally similar potentially harmful proteins, along with a high content of fermentable carbohydrates. These carbohydrates, which are partially absorbed in the gastrointestinal tract and subsequently fermented by gut microbiota, have been shown to offer benefits in managing functional gastrointestinal disorders in humans. Likewise, a study by Lewis et al. reported that dogs fed a low-protein, high-carbohydrate (LPHC) diet had an increased abundance of bacteria associated with SCFAs production [77,78]. In another study, sixty-four dogs from two breeds were divided into two groups: 32 received a high-protein/low-carbohydrate (HP/LC) diet and 32 received a low-protein/high-carbohydrate (LP/HC) diet. Stool samples were collected before and after the dietary interventions. In this dataset, the *Prevotella*-to-*Bacteroides* ratio was higher under the baseline and LP/HC diets compared to the HP/LC diet, a pattern similar to that observed in humans. A higher *Prevotella*-to-*Bacteroides* ratio is often associated with beneficial metabolic outcomes, such as improved fiber fermentation and SCFAs production, suggesting that these diets may promote a healthier gut microbial profile [79].

Beyond skin disorders, diet also plays a critical role in the development of obesity and diabetes in dogs [14,32].

Dietary changes with the use of probiotics, prebiotics and postbiotics can promote benefits on several levels, as will be discussed below.

### 3.2. Probiotics

**Figure 2:**
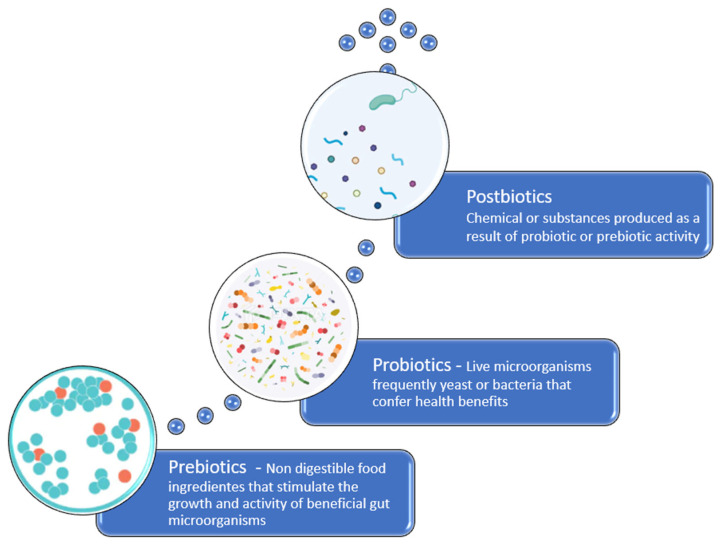
Schematic representation illustrating the definitions of prebiotics, probiotics, and postbiotics.

Probiotics are live microorganisms that are administered to a patient with the aim of inhibiting the growth and adhesion of pathogens to the intestinal epithelium, supporting mucosal barrier function, and mitigating antimicrobial-induced disturbances (Figure 2). The gut microbiota generates a wide range of metabolites and small reactive molecules through the metabolism of dietary components and other exogenous substances. These metabolites—such as SCFAs, tryptophan (Trp) derivatives, serotonin, indole, γ-aminobutyric acid (GABA), and bile acids (BAs)—act as signaling molecules in host–microbiota interactions and play a crucial role in regulating host metabolism. Growing evidence suggests that probiotic supplementation can influence the production and secretion of these bioactive compounds, which engage in bidirectional communication along the gut–brain, gut–lung, gut–liver, and gut–heart axes. This interaction contributes to improving the host’s health and can help prevent or lessen various diseases [80]. Some probiotics can produce bacteriocins that interfere with the quorum-sensing systems of intestinal pathogens, reducing the host’s susceptibility to infection, enhancing IgA production, improving nutrient absorption, and downregulating the secretion of pro-inflammatory cytokines [80]. Certain probiotic strains also exhibit potent immunomodulatory properties at the skin level, with the most robust evidence supporting their efficacy in gut-related disorders such as diarrhea, irritable bowel syndrome, and *C. difficile* infection [8,14,76]. Koziel et al. demonstrated for the first time that the probiotic strain *Escherichia coli Nissle* (EcN) 1917 exerts a dose-dependent protective effect against acute gastric lesions induced by stress exposure. These findings are consistent with previous reports indicating that probiotics confer protective activity not only in the lower gastrointestinal tract (e.g., the colon), but also in the gastric mucosa, where they help mitigate acute mucosal injury [81].

Probiotics can serve as adjuncts to antimicrobial and anticancer therapies, enhancing treatment efficacy, potentially accelerating recovery, and reducing the required dosage. Evidence suggests that probiotics are more effective when administered within the first two days of initiating antimicrobial therapy. However, there is currently no compelling evidence supporting the superior efficacy of any specific probiotic strain, formulation, delivery method (e.g., drink or capsule), or dosage [82].

Several randomized controlled trials involving 39 adults indicate that probiotics can have a beneficial effect on various aspects of metabolic syndrome, including blood pressure, glucose metabolism, and blood lipid profiles, as well as on improving inflammatory biomarkers. Wastyk et al. state that the response to probiotic supplementation in individuals with metabolic syndrome may be influenced by dietary factors. Their study demonstrated that probiotic intake led to improvements in triglyceride levels and diastolic blood pressure compared to the placebo group [76]. Kang et al. proved that probiotic supplementation influenced the proliferation of commensal bacteria and enhanced glycolysis, leading to the activation of pyruvate metabolism. This activation is linked to propionate metabolism and promotes bacterial fatty acid production through dopamine and carboxylic acid–related pathways. As a result, ATP synthesis and overall energy metabolism in the host were increased, which helped prevent lipid accumulation and restore stability to the fecal microbiota. Consequently, this led to a reduction in systemic inflammation induced by a high-fat diet [83].

Microorganisms of the genus *Lactobacillus* are commonly used as probiotics [84]. Regarding atopic dermatitis, Kim et al. investigated the effects of orally administering *Lactobacillus acidophilus* KBL409 over a 28-day period, in an AD—induced mouse model. The treatment significantly reduced various clinical symptoms of AD, including erythema/haemorrhage, scaling/dryness, oedema, and excoriation/erosion. Furthermore, it resulted in lowered serum IgE concentrations and diminished immune cell infiltration in the skin [85], thereby reducing SCORAD (Scoring Atopic Dermatitis Index) values and even lessening the likelihood of AD onset [34]. Supplementation with *Lactobacillus reuteri* in mice improved skin health by increasing dermal thickness, hair follicle activity, increasing production of sebaceous cells, and decreasing skin acidity, resulting in thicker and shinier coats compared to mice that did not receive the probiotic [62].

Probiotic therapy aimed at restoring a healthy intestinal and/or skin microbiota may serve as an important adjuvant treatment to handle various inflammatory skin diseases, such as AD [43], acne, psoriasis, and rosacea. Ongoing research in this area holds promising potential for advancing scientific understanding and expanding therapeutic options across various fields of dermatology, grounded in the emerging concept of the gut-brain-skin axis [86]. A 12-week randomized, double-blind, placebo-controlled clinical trial evaluated the effects of a probiotic supplement containing *Lacticaseibacillus rhamnosus* (CECT 30031) and *Arthrospira platensis* in patients aged 12 to 30 years with acne vulgaris (AV). The probiotic group showed a significant reduction in non-inflammatory lesions compared to the placebo group [87]. Likewise, oral supplementation with *Lactobacillus acidophilus* and *Lactobacillus bulgaricus* reduced acne in 80% of the 300 participants, with the most noticeable benefits seen in those with inflammatory acne lesions [74]. However, Bae et al. reported a case of canine infective endocarditis caused by *Bacillus amyloliquefaciens*, a Gram-positive bacterial species commonly used as a probiotic in both humans and animals. While there is no direct evidence linking the isolated bacteria to probiotic use in this case, caution is advised when administering spore-forming *Bacillus* species as probiotics, particularly in hospitalized or immunocompromised individuals [88].

Collectively, many reviewed studies underscore the promising role of probiotic-based topical formulations as innovative therapeutic strategies in dermatology, with the potential to improve patients’ quality of life [43]. These formulations have demonstrated beneficial effects, including the promotion of ceramide and essential lipid production, which reinforces skin barrier function. Moreover, probiotics compete with pathogenic species, colonize the skin, interact beneficially with the resident microbiota, and modulate the local immune response. In addition, they exert anti-inflammatory effects by downregulating pro-inflammatory cytokines and upregulating anti-inflammatory mediators [89].

The use of probiotics has become increasingly important in the management of infected wounds, as they promote the healing process by competing with pathogenic microorganisms for nutrients and binding sites on the host mucosa. This effect is mediated through the production of lactic acid and bacteriocins, as well as the recruitment of macrophages and PMNs, which help control infection and support tissue repair [44]. Another study demonstrated that a lysate of *Lactobacillus reuteri* enhances wound repair, increases the number of oxytocin-positive cells in the paraventricular nucleus (PVN) of the hypothalamus in mice, and is associated with reduced corticosterone levels, suggesting a stress-modulating effect [90].

Topical application of probiotics in wound healing has shown promising results in some studies, demonstrating beneficial effects such as enhanced granulation tissue formation, increased collagen deposition, and stimulation of angiogenesis. However, other studies have reported no significant improvement, highlighting variability in therapeutic outcomes. Topical probiotic interventions in dermatology are still in the early stages of investigation, and further research is needed to establish their efficacy, elucidate underlying mechanisms of action, and, most importantly, assess their safety. Potential risks include antimicrobial resistance gene transfer, allergic reactions, and bacteremia, particularly in immunocompromised individuals, including infants, pregnant women, and the elderly [91].

Kambel et al. investigated the use of probiotics as programmable bacteria and delivery vehicles for therapeutic nanobodies and viral antigens, improving outcomes in viral infections and modulating the immune response [92]. In 2020, Jiang et al. indicated that probiotic interventions may reduce the incidence of AD and relieve symptoms in children, particularly in infants and children aged ≥1 year with AD. Moreover, mixed-strain probiotic interventions appeared to provide greater preventive and therapeutic benefits [93].

Additionally, future research into single-cell engineered live biotherapeutic products (eLBPs) focuses on genetically and/or metabolically modified microorganisms (“designer probiotics”) intended to deliver targeted therapies directly to disease sites. Using bacteria as synthetic biology platforms, this approach has shown promise in treating various skin conditions, including wound healing, skin regeneration, and cancer [94].

### 3.3. Prebiotics

Prebiotics are non-digestible dietary substrates that specifically stimulate the proliferation and metabolic activity of advantageous microbial populations within the gastrointestinal tract thus conferring benefits upon the host’s health [31] (Figure 2). The beneficial effects are based on selecting the growth and so increasing the number of bacteria already resident in the colon [14,95]. Most prebiotics research has focused on oligosaccharides, the fructans, including frutooligosacharides (FOS), Inulin, and galactooligosacharides (GOS). Inulin and FOS are now widely included in commercial pet foods [14,95]. Prebiotics provide fermentable substrates that selectively support the growth of beneficial gut microorganisms, including *Bifidobacterium* and *Bacteroides*. They enable the establishment of bifidogenic populations while limiting the expansion of pathogens such as *C. difficile*. Beyond shaping the microbiota, prebiotics influence host physiology by regulating immune responses, enhancing cognitive and neurological functions, and improving the absorption of essential minerals. They are also associated with lowering circulating lipid levels and mitigating insulin resistance, thereby contributing to overall metabolic and cardiovascular health [96,97].

One study investigated the effect of red ginseng dietary fiber on the gut microbiota and host response in dogs. Red ginseng contains bioactive compounds such as ginsenosides and saponins, which contribute to its health-promoting effects, including anti-inflammatory, anticancer, and anti-obesity properties. Red ginseng dietary fiber modified the canine gut microbiota by increasing microbial diversity, enriching SCFA-producing bacteria, reducing potential pathogens, and enhancing the complexity of microbial interactions. These findings suggest that red ginseng-derived dietary fiber may support canine gut health by modulating the microbiota, highlighting its potential as a prebiotic [98].

Inulin is a well-characterized prebiotic known to modulate the gut microbiota and exert lipid-lowering effects. Consistent with previous research, inulin supplementation in hens significantly reduced serum and hepatic total cholesterol (TC) and triglyceride (TG) levels in models of metabolic-associated fatty liver disease (MAFLD) [99].

### 3.4. Postbiotics

Postbiotics represent a novel category of biotics comprising bioactive compounds produced as metabolites and structural derivatives of microorganisms, which offer several advantages over other biotics (Figure 2). They are considered safe, have a long shelf life, are easy to administer, pose minimal risk, and are generally more appealing than probiotics. In addition, they are easily produced, present no risk of horizontal gene transfer related to AMR, are suitable for encapsulation, and can be targeted to specific sites within the host [100].

Postbiotics are typically categorized based on their molecular weight and structural attributes, encompassing non-viable microbial cells, metabolic byproducts, and structural components originating from the gut microbiota [100]. Broadly, they are divided into two main groups: cell-associated and cell-free postbiotics. The former includes inactivated microorganisms, cell lysates, and various microbial fragments, while the latter comprises cell-free supernatants (CFS), cell-free spent media (CFSM), and a wide array of secreted metabolites.

These compounds span a diverse range of bioactive molecules such as SCFAs, organic acids, vitamins, flavonoid- and phenolic-derived metabolites, exopolysaccharides (EPS), lipoteichoic acids (LTAs), and terpenoid-based compounds, all of which exert physiological effects in both humans and animals [101].

Butyrate, a well-characterized SCFA, serves as a key example of a postbiotic due to its dual role as an energy substrate and regulator of intestinal development, epithelial cell differentiation, and immune modulation. Prolonged butyrate supplementation has been shown to prevent diet-induced obesity and metabolic disturbances by suppressing appetite and enhancing thermogenesis in brown adipose tissue (BAT). These effects are mediated in part by increased sympathetic nervous system activity and require intact vagal signaling, as demonstrated by their absence following subdiaphragmatic vagotomy [102]. Furthermore, several postbiotic interventions have demonstrated efficacy in reducing cholesterol levels, often showing superior outcomes compared to untreated controls [86]. Experimental models have extensively studied various postbiotic compounds, including SCFAs, EPS, bacterial cell wall fragments, and lysates, for their therapeutic potential [103].

Postbiotics have well-defined chemical structures capable of modulating the composition and activity of the host’s native gut microbiota, thereby conferring health benefits such as anti-aging and anti-inflammatory effects. These effects were confirmed by Kim, who investigated the postbiotic MB-2006, produced by fermenting *Smilax china* L. with *Lactobacillus* strains, and demonstrated that it exhibits significant anti-inflammatory and immunoregulatory properties in HaCaT keratinocytes stimulated with TNF-α and IFN-γ [104]. They also contribute to strengthening the skin barrier, promoting hydration, and maintaining an acidic pH—all of which are essential for preventing microbial overgrowth. These actions support functional outcomes such as UV protection, enhanced wound healing, and the preservation of skin homeostasis. Additionally, postbiotics influence immune function, neurohormonal signaling, lipid metabolism, and behavior, further highlighting their systemic health benefits. Postbiotics reduce the risk of infections and adverse immune reactions, making them particularly beneficial for individuals with weakened immune systems or compromised skin barriers [100]. Postbiotics have been shown to slow tumor growth and cancer progression while maintaining intestinal integrity. In addition, several widely used oral probiotics produce bacteriocins with notable anticancer properties, underscoring the need for further research into the therapeutic potential of purified bacteriocin compounds. Conventional anticancer therapies are often limited by drug resistance and non-selective cytotoxicity, which can lead to adverse side effects. Bacteriocins represent a promising alternative, and their efficacy may be further enhanced through conjugation with nanoparticles, enabling synergistic drug delivery and reduced dosing [105]. In addition, they exert therapeutic mechanisms as anti-diabetic and anti-inflammatory agents [106].

A study using heat-treated *Lactobacillus* strains demonstrated a significant reduction in the severity of AD in children aged 4–30 months. Compared with live probiotics, heat-treated strains offer a safe alternative, enhancing epithelial barrier function, downregulating inflammatory markers, and protecting against pathogens. These findings suggest a potential therapeutic role in dermatological conditions such as AD and provide further evidence of the gut–skin axis [107].

### 3.5. Stress Reduction

Stress has become an integral aspect of modern life, particularly in developed societies [108]. Emotional stress can alter the intestinal microbiota, increase intestinal permeability, and contribute to systemic inflammation. Recent evidence indicates that both the skin and the gut act as immediate stress sensors and target organs affected by systemic stress responses. The gut influences brain function and behavior, and vice versa, including stress-related behaviors, thereby contributing to the development of pathological conditions such as anxiety and depression [109,110,111,112]. In two cohorts of healthy adults stratified by stress levels, stress accounted for approximately 5% of the variance in gut microbiota composition. Individuals with lower perceived stress exhibited greater microbial diversity compared to those with higher perceived stress [112]. The gut microbiota produces a variety of metabolites that influence brain function. SCFAs, such as propionate, enhance memory, support neural plasticity, and protect the blood–brain barrier, while also modulating immune responses and neuroinflammation. Specific bacterial taxa contribute directly to neurotransmitter production: *Lactobacillus* and *Bifidobacterium* produce acetylcholine and GABA, while *Streptococcus*, *Enterococcus*, and *Escherichia* generate serotonin, dopamine, and norepinephrine. In addition, gut microbes synthesize neuroprotective vitamins (K, B2, B9, B12) and regulate tryptophan metabolism, thereby influencing host availability of serotonin, melatonin, and indole [111]. The gut–brain axis may also play a critical role in the relationship between the microbiota and AD, as individuals with AD are at increased risk of psychological comorbidities, including anxiety, depression, and reduced psychological well-being [32].

Numerous studies support the existence of the gut–brain–skin axis and demonstrate that psychological stress contributes to intestinal dysbiosis [109]. During stress, the body releases a cascade of hormones—including corticotropin-releasing hormone (CRH), glucocorticoids, and catecholamines such as epinephrine—to facilitate physiological adaptation. However, these hormones can disrupt gut microbiota composition and influence systemic immune and inflammatory responses [108]. The intestinal microbiota can interact with the hypothalamus and pituitary gland through neurological and immunological pathways [113]. Varian et al. demonstrated that mice administered a probiotic strain exhibited elevated oxytocin levels and significantly improved wound healing. This effect was mediated by oxytocin receptors in fibroblasts and keratinocytes, leading to reduced corticosterone levels and accelerated wound closure [90].

### 3.6. Fecal Microbial Transplantation (FMT)

Fecal Microbiota Transplantation (FMT) involves the administration of homogenized fecal suspensions enriched with a healthy donor’s commensal microbial consortia into a recipient’s gastrointestinal tract, with the objective of re-establishing eubiosis and restoring the structural and functional integrity of the gut microbiota [31]. FMT has been used to successfully treat recurrent *C. difficile* infection with cure rate higher than 85.6%. There are preliminary indications to suggest that it may also carry therapeutic potential for other conditions such as inflammatory bowel disease, allergic disease, diabetes [114], obesity, metabolic syndrome and functional gastrointestinal disorders [115].

FMT presents certain limitations, including significant interindividual variability in microbiota composition, which complicates the distinction between healthy and dysbiotic profiles. Additionally, there is a potential risk of transferring microorganisms with pathogenic potential, particularly those harboring antimicrobial resistance genes. To minimize and prevent adverse events associated with FMT procedures, the implementation of rigorous donor screening protocols is strongly recommended. Accurate characterization of the microbiota in both healthy and dysbiotic states, along with prospective studies assessing the microbiome prior to disease onset, is essential for establishing a causal relationship between dysbiosis and disease [1].

The transplantation can be administered through several routes, each with distinct advantages and limitations. The upper gastrointestinal (GI) route, including esophagogastroduodenoscopy (EGD) and nasogastric or nasoduodenal tubes, is suitable for patients with colonic inflammation but is limited by risks of aspiration, patient discomfort, and the inability to assess colonic mucosa or obtain tissue samples. Colonoscopy remains the most effective method for full colon recolonization due to its capacity to deliver fecal material throughout the colon and perform mucosal evaluation; however, it is a costly, invasive, and high-risk procedure. Retention enemas are less invasive and more cost-effective, although they only reach the distal colon. Oral capsule-based FMT is emerging as a well-tolerated, non-invasive option with high patient adherence, but it is limited by high production costs and the need to ingest a large number of capsules [114].

Studies on recurrent CDI have primarily involved patients exposed to multiple antimicrobial courses, leading to progressive loss of microbial diversity. This dysbiosis is marked by loss of *Bacteroides*, a reduction in *Firmicutes*, and a surge in *Proteobacteria*. On average, patients experienced five CDI episodes over one year before undergoing fecal microbiota transplantation. Colonoscopic FMT rapidly restores microbial community structure—normalization occurs within 24 h, with re-establishment of *Bacteroides* and *Firmicutes* dominance. The engraftment of donor microbiota is sustained for months to years, indicating long-term correction of dysbiosis in refractory CDI [116].

Multiple studies have demonstrated that FMT is a safe and effective strategy for AD in both humans and dogs. In a clinical study, Mashiah et al. evaluated FMT in adults with moderate to severe AD and observed significant improvement in clinical symptoms after four treatment sessions, with no adverse events reported. Despite the limited sample size, these findings provide early clinical evidence supporting the involvement of gut microbiota in AD pathogenesis and the therapeutic potential of FMT [117]. Washed microbiota transplantation (WMT) successfully relieved clinical symptoms in an adolescent with atopic dermatitis, accompanied by improvements in serum eosinophil ratio (EOSR), TNF-α, IL-6 levels, and restoration of both skin and gut microbiota. This case report offers clinical evidence supporting WMT as a potential therapeutic approach for AD, likely through modulation of the skin microbiota and inflammatory pathways via the gut–skin axis [118]. Similarly, Kim et al. were among the first to demonstrate FMT’s efficacy in an animal model, showing that it relieved allergy-like symptoms in mice by restoring gut microbiota composition and Th1/Th2 immune balance [119,120].

However, patients with AD often exhibit impaired gut barrier function, which may increase their susceptibility to potential risks associated with FMT [120]. Further research is required to fully understand its mechanisms and long-term efficacy.

## 4. Conclusions and Future Perspectives

While modern advancements—such as improved hygiene, vaccination programs, and the widespread use of antimicrobials—have markedly reduced the incidence and severity of infectious diseases, they have also inadvertently disrupted the gut microbiota. These changes may compromise its protective functions, including pathogen resistance, immune modulation, and maintenance of mucosal integrity. Preserving or restoring a diverse and resilient microbial ecosystem—one that effectively occupies metabolic niches and prevents pathogen colonization—has become a central focus in the fight against infectious and immune-related diseases. Beneficial commensals and their metabolic byproducts, capable of stimulating trained innate immunity while maintaining immune homeostasis, represent a promising avenue for the development of innovative therapeutic approaches.

Microbiota-targeted interventions have shown promise in managing immune-mediated conditions such as AD. They act by modulating systemic and cutaneous inflammation through the gut–skin axis and promoting immune homeostasis. As the onset and progression of AD are closely associated with alterations in the gut microbiota, with reduced levels of beneficial bacteria commonly observed in patients, microbiota manipulation may serve as an effective strategy to restore intestinal function and replenish these beneficial microorganisms. Such interventions can reshape the gut microbial environment, help maintain microbial balance, and regulate systemic immune responses. Overall, microbiota-based interventions may alleviate the clinical manifestations of AD by influencing microbial composition, metabolic activity, and immune regulation. Evidence indicates that they can reduce SCORAD (Scoring Atopic Dermatitis) scores and lower the risk of AD development. However, outcomes remain inconsistent, likely due to environmental, dietary, and methodological variations. Future studies with larger subjects and more rigorous experimental designs are required to confirm the efficacy of probiotics and related strategies in AD management.

Controlling AD through microbiota manipulation also has the advantage of reducing the use of antimicrobials, decreasing selection pressure on antimicrobial-resistant bacterial strains, helping to lower AMR. Taken together, microbiome-targeted approaches represent a paradigm shift in the prevention and treatment of both infectious and immune-mediated diseases. Their sustainable, resistance-limiting potential supports integration into clinical, veterinary, and environmental practices, in line with the One Health framework. As such, they hold significant promise for addressing the global AMR crisis while promoting long-term human and animal health.
